# How to Improve the Care of Women after Childbirth in the Rooming-in Unit: A Prospective Observational Study

**DOI:** 10.3390/ijerph192316117

**Published:** 2022-12-02

**Authors:** Anna Prokopowicz, Bartłomiej Stańczykiewicz, Izabella Uchmanowicz, Mariusz Zimmer

**Affiliations:** 1 Department of Nursing and Obstetrics, Wroclaw Medical University, 51-618 Wroclaw, Poland; 2 Department of Psychiatry, Wroclaw Medical University, 50-367 Wroclaw, Poland; 3 2nd Department of Gynaecology and Obstetrics, Wroclaw Medical University, 50-556 Wroclaw, Poland

**Keywords:** anxiety, pain, rooming-in, patient-oriented outcomes, newborn, maternity

## Abstract

Rooming-in is the WHO-recommended care system for mothers in the puerperium and their babies. This system allows the newborn to stay with the mother in the same room, 24 h a day. We aimed to investigate the need to entrust a newborn (NEN) in the care of maternity rooming-in staff during the COVID-19 pandemic, and its relationship to pain, anxiety, and blood loss after delivery. A prospective study of 200 adult women in the maternity ward operating in the rooming-in system focussed on NEN in the care of maternity rooming-in staff on the first (T1) and the second day of puerperium (T2). Women who declared having NEN were compared with women without NEN for anxiety, pain, and a drop in haemoglobin in the blood after delivery. In T1, 34% and in T2, 27% of women felt NEN in the care of maternity rooming-in staff. The NEN of women after a cesarean section was higher on both days than the NEN of women after vaginal delivery. Women with NEN had higher levels of pain, state anxiety, and higher levels of postpartum anxiety than women without NEN. Further research should be warranted to investigate whether women who give birth in hospitals that satisfy the NEN in the care of maternity rooming-in staff in their rooming-in units experience less pain and anxiety in comparison to those who give birth in hospital units without such a possibility and whether this factor is an important element in reducing anxiety and pain during puerperium.

## 1. Introduction

Rooming-in is a hospital practice that allows the mother to stay in the same room with her newborn baby 24 h a day after giving birth [1]. This system was implemented as a result of the global initiative of the Baby-Friendly Hospital Initiative, whose primary goal was to implement evidence-based maternity care to support and enhance breastfeeding [2], and to build a mother-child bond [3,4,5]. Hospitals vary in implementing a full rooming-in system in maternity units. Some facilities are still using a nursery, part of the mixed system, and part of the total rooming-in without a nursery [6,7]. In the rooming-in system, the newborn is in the room around the clock with the mother, who, with the support and care of the staff, carries out most of the nursing activities for the newborn on her own.

The rooming-in system promotes the participation of relatives being close and aids in caring for the mother-child couple [8]. Nakić Radoš et al. showed that women who received less support from their relatives had a higher level of anxiety as a state in the puerperium [9]. It can be assumed that a similar corelation may be observed during the COVID-19 pandemic. Due to the current epidemiological situation related to the pandemic, which influenced the prohibition of visiting hospitals, women cannot count on family support in caring for a newborn in the first days of puerperium at all. This situation has not changed over time since the threat of new waves of mutations in the SARS-CoV-2 virus is periodically spreading throughout the world. A study of Polish women in childbirth during the pandemic showed a relationship between the occurrence of postpartum depression and the lack of support from relatives [10]. In this study, higher levels of depression were also associated with higher levels of pain experienced [10]. Higher levels of pain in the puerperium reduce the mobility of a women and her ability to care for herself [11].

The weakening of the woman’s emotional and physical condition can also be associated with blood loss in childbirth [12,13]. Several studies have shown that some women who do not have a clinical history of postpartum haemorrhage, have changes in blood count on the second day postpartum, indicating abnormal peripartum blood loss [14,15]. Haemorrhage studies define undiagnosed abnormal postpartum blood loss when the difference in a haemoglobin concentration before and after delivery is greater than or equal to 2 g/dL [15,16].

Postpartum fatigue is a common phenomenon. A longitudinal study by Rychnovsky [17] showed that 62% of women experienced moderate fatigue on the second day after delivery, while 18.3% of women experienced severe fatigue. Another study found that 38.8% of mothers experienced severe fatigue 10 days after giving birth despite having received the support of their partner. The reported fatigue was influenced by depression and anxiety, as well as problems with sleep and breastfeeding [18].

Due to their psychophysical condition, women can differ in terms of the need to place a newborn under the care of the staff in the ward operating in the rooming-in system. The initial nursing assessment aims to collect information about the woman’s physical and emotional condition and individual needs. The collected data are used to recognise the general condition and mental condition of the woman, as well as to set up emergency interventions [19].

The aim of our study was to investigate the need to entrust a newborn (NEN) in the care of maternity rooming-in staff in puerperial women in a rooming-in unit during the COVID-19 pandemic. The second objective was to investigate the association of NEN with pain, anxiety, and blood loss after delivery. The third objective of the study was to compare the group of mothers declaring having NEN in the care of maternity rooming-in staff with the group of mothers without the above need in terms of pain, anxiety and blood loss after delivery, and to identify the cut-off point on the pain and anxiety scale for women declaring having NEN.

## 2. Materials and Methods

### 2.1. Population and Settings

The study was conducted in the rooming-in maternity ward of the tertiary hospital. The ward is equipped with a newborn observation room adjacent to the patient observation room where puerperas stay on Day 0 (the first 24 h after delivery) after cesarean section (CS) or regardless of the day of puerperium, patients requiring strict supervision due to their physical condition (both after vaginal delivery and after CS). Patient observation room staff look after children whose mothers were not yet mobilised. After the observation period and after mobilisation, most often in the first 24 h after delivery, women after CS are transferred with the newborn to the maternity ward operating in the rooming-in system. Women after physiological vaginal delivery are transferred from the delivery block directly to rooming-in. 

The study included 200 women on the first (24 h from childbirth to 48 h) and on the second day (48 h to 72 h) after delivery, staying with their baby at the rooming-in ward. We enrolled adult women who gave their informed consent to the study, gave birth at term (i.e., ≥37 weeks of gestation) to a single newborn in good condition (from 8–10 Apgar points), both by vaginal delivery and CS. The inclusion criterion was the good clinical condition of the mother after childbirth (the woman was mobilised and without any diagnosed mental, psychological, and orthopaedic dysfunction as well as negative for SARS-CoV-2) and normal blood loss during delivery as clinically assessed by an operator (no haemorrhage). The data were collected from December 2020 to April 2021.

Data on obstetric condition, woman’s medical history, and health condition of the newborn were obtained from the woman’s and the newborn’s medical history. The study was confidential. Access to the collected data was secured. Each of the study participants was given an ID number, which allowed data anonymisation.

### 2.2. Scales

#### 2.2.1. Need to Entrust a Newborn under the Care of the Staff

The need to put a newborn in the care of the staff was examined with a single-question questionnaire developed by the authors: ‘In my current situation, I have a need to entrust my newborn baby in the care of staff for some time.’ The responses were given on a 6-point Likert scale (1—‘I strongly disagree’, 2—‘I disagree’, 3—‘I disagree a little’, 4—‘I agree a little’, 5—‘I agree’ and 6—‘I strongly agree’). For the purpose to differentiate the women in terms of NEN, the answers from 1 to 3 defined the group of women without the NEN (NEN_low), and the answers from 4 to 6 defined the group of women declaring having NEN (NEN_high).

#### 2.2.2. Anxiety Measurement

The State-Trait Anxiety Inventory (STAI) [20] was developed as an original tool and adapted for use in the Polish population by the Spielberger team. It consists of two separate scales: X-1 for state anxiety (STAI-S) and X-2 for trait anxiety (STAI-T). Both scales consist of 20 questions. The total continuous scores range from a minimum of 20 to a maximum of 80. The higher the total score, the higher the level of anxiety. In our study, Cronbach’s alpha for state anxiety (STAI-S) on the first day (T1) was 0.956 and on the second day (T2), it was 0.958. Cronbach’s alpha for trait anxiety (STAI-T) was 0.850.

#### 2.2.3. Numerical Rating Scale for Anxiety

The Numerical Rating Scale for Anxiety (NRS-A) [21,22] was used to measure state anxiety. The score ranged from 0 to 10, where 0 denotes no anxiety and 10 represents the greatest anxiety imaginable. Despite its simplicity, it has been shown to be valid and reliable. 

In addition, the women were asked to provide subjective levels (on a scale of 0 to 10) of specific anxiety on the second day, such as anxiety of self-care during the day, self-care at night, before a child’s crying, before breastfeeding, before asking staff for help, before contracting COVID-19.

#### 2.2.4. Numerical Rating Scale for Pain

The Numerical Rating Scale for Pain (NRS) was used to measure the level of pain, as it is commonly used to measure pain. The score ranged from 0 to 10, where 0 denotes no pain and 10 represents the greatest pain imaginable. Despite its simplicity, it has been shown to be valid and reliable [23].

### 2.3. Measurement of the Haemoglobin Concentration in the Blood

Each woman had a blood count performed on the day before delivery and on the second day after delivery. The haemoglobin concentration on the second day was subtracted from the haemoglobin concentration before delivery, thus obtaining the difference (in g/dL) in the haemoglobin concentration in the blood.

### 2.4. Study Protocol

After giving their informed consent to participate in the study, the women were asked to complete questionnaires in two measurements: on the first day (T1) and the second postpartum day (T2). On both days, the women completed the NRS, NRS-A, STAI-S-X1, and NEN scales. Additionally, they completed the STAI-T-X2 questionnaire (on T1), and specific perinatal anxiety on an NRS-A from 0 to 10 (on T2). The woman’s blood count was taken on the day before delivery and the second day after delivery.

### 2.5. Statistical Analyses

The IBM SPSS Statistics 26 program (IBM Corp., Armonk, NY, USA) was used for statistical analysis. The obtained values of the statistics showed that all variables, apart from the difference in blood haemoglobin concentration, showed significant discrepancies from the normal distribution. The relationship between the variables was calculated using Spearman’s correlation coefficient. The Mann–Whitney U test and the Student’s *t*-test were used for comparison of independent variables, and the Wilcoxon’s test for comparison of dependent variables. The thresholds to distinguish anxiety and pain from the absence or presence of NEN (defined by the NEN reference cut-off point of 3) were determined from the receiver operating characteristic (ROC) curves and the associated area under the ROC curve (AUC). The significance of the measurements was assumed for the value of *p* < 0.05.

## 3. Results

There were no missing data in the questionnaires. On the second day, two missing blood counts were observed.

Of the 200 women with a median age of 32 years (IQR = 5; range: 21–43) surveyed, 56% (*n* = 112) of the women underwent CS, and 44% (*n* = 88) delivered naturally. Overall, 49.5% (*n* = 99) of the mothers were primiparous, and 50.5% (*n* = 101) of the mothers were multiparous. On day one, 34% (*n* = 68) of women declared having NEN (NEN_high_T1), and on day two, 27% (*n* = 54).

On both days, the median NEN was 2 (IQR = 3; range: 1–6). A high positive relationship was demonstrated between NEN_T1 and NEN_T2 (rho = 0.679; *p* < 0.001). The NEN on the second day was lower compared to the first day (Mrank_NEN_T2_ = 41.02 vs. Mrank_NEN_T1_ = 43.93; Z [2.199] = 2.382; *p* < 0.05).

On both days, there were no differences in NEN between the group of primiparous women versus multiparous women, but the NEN of women after CS was higher on both days than the NEN of women after vaginal delivery (Table 1).

### 3.1. NEN and Pain, Anxiety, and Haemoglobin

The conducted analysis showed statistically significant positive relationships between NEN and anxiety and pain on both days. It turned out that the higher the woman’s pain and anxiety, the greater the NEN. The strongest relationship of NEN was observed with state anxiety (on T1: rho = 0.44; *p* < 0.001 and on T2: rho = 0.45; *p* < 0.001). However, there was no evidence of a relationship between NEN and the decrease in haemoglobin concentration in the blood after delivery (Table 2).

### 3.2. Comparison of Pain, Anxiety, and Difference in Haemoglobin Concentration in Women in Groups NEN_Low vs. NEN_High in T1 and T2

The Mann–Whitney U test showed on both days (T1 and T2) that women who felt NEN (NEN_high) had higher levels of pain and anxiety as a state than women without NEN (NEN_low). There were no significant differences between the groups in the level of trait anxiety (Table 3).

The Student’s *t*-test showed no significant differences in both measurements in the decrease in the haemoglobin concentration (g/dL) in the blood of women between women who had NEN and those who had NEN_low (on T1: M_NEN_low_ = 1.522; SE_NEN_low_ = 1.007 vs. M_NEN_high_ = 1.500; SE_NEN_high_ = 0.900; t [2.197] = 0.155; *p* > 0.05 and on T2: M_NEN_low_ = 1.442; SE_NEN_low_ = 0.943 vs. M_NEN_high_ = 1.711; SE_NEN_high_ = 1.045; t [2.197] = 1.642; *p* > 0.05).

### 3.3. Relationship of Specific Anxiety with NEN and Comparison of Specific Anxiety of Women with and without NEN on the Second Day of Puerperium

The conducted analysis showed statistically significant positive relationships between NEN and specific anxiety (Table 4). The strongest positive relationship of NEN was observed with anxiety about self-care at night (rho = 0.516; *p* <0.001) and during the day (rho = 0.459; *p* < 0.001). The weakest positive correlation of NEN was demonstrated with the anxiety of developing COVID-19 (rho = 0.158; *p* < 0.05).

Women who felt NEN had a higher level of self-care anxiety (day and night), crying the baby, breastfeeding, and asking the staff for help than women who did not have NEN. There were no significant differences between the groups in the level of anxiety of developing COVID-19 (Table 5).

### 3.4. Cut-Off Point on the NRS, NRS-A and STAI-S Scale for the High NEN (According to NEN = 3)

**Figure 1:**
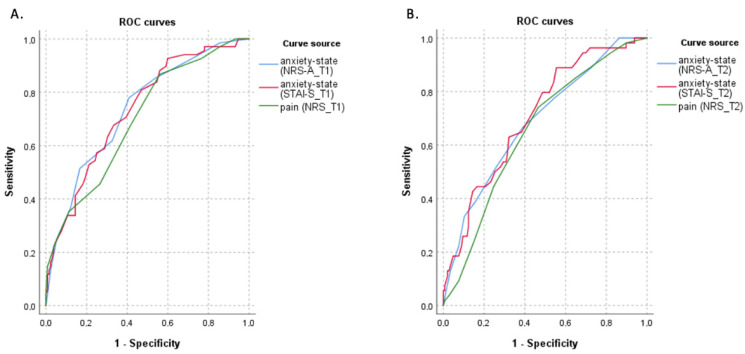
1. ROC curves. (**A**). Calculated for NRS_T1 (AUC = 0.699; *p* < 0.001), for NRS-A_T1 (AUC = 0.737; *p* < 0.001) and for STAI-S_T1 (AUC = 0.709; *p* < 0.001) using NEN_T1 where a score greater than 3 was selected as an indicator of the presence of NEN on T1. The NRS cut-off value of 3.5/10 reflected the best combination of sensitivity (87%) and specificity (44%). The NRS-A cut-off value of 3.5/10 reflected the best combination of sensitivity (78%) and specificity (59%). The STAI-S cut-off value of 42.5/80 reflected the best combination of sensitivity (68%) and specificity (67%). (**B**). Calculated for NRS_T2 (AUC = 0.654; *p* < 0.001), for NRS-A_T2 (AUC = 0.689; *p* < 0.001) and for STAI-S_T2 (AUC = 0.709; *p* < 0.001) using NEN_T2 where a score greater than 3 was selected as an indicator of the presence of NEN on T2. The NRS cut-off value of 3.5/10 reflected the best combination of sensitivity (74%) and specificity (53%). The NRS-A cut-off value of 3.5/10 reflected the best combination of sensitivity (67%) and specificity (61%). The STAI-S cut-off value of 37.5/80 reflected the best combination of sensitivity (89%) and specificity (44%).

The analysis of the ROC curve for 200 women suggested a value of 3.5/10 on the NRS in both measurements (on T1: AUC = 0.699; *p* < 0.001 and on T2: AUC = 0.654; *p* < 0.001) and a value of 3.5/10 on the NRS-A (in T1: AUC = 0.737; *p* < 0.001 and in T2: AUC = 0.689; *p* < 0.001) as the threshold for having NEN (defined by the reference cut-off point for NEN of 3). For the STAI-S scale, the analysis of the ROC curve indicated the value of 43.5/80 on the first day of the puerperium (on T1: AUC = 0.734; *p* < 0.001) and 37.5/80 on the second day of puerperium (on T2: AUC = 0.709; *p* < 0.001) (Figure 1).

## 4. Discussion

In our study, less women felt NEN in the care of maternity rooming-in staff on the first day than on the second day after delivery (34% vs. 27%). The NEN of women after CS was higher on both days than the NEN of women after vaginal delivery. In addition, women with NEN had higher levels of pain, state anxiety, and higher levels of postpartum anxiety than women without NEN. Other reports investigated overall anxiety; however, this study focused on postpartum anxiety associated specifically with self-caring for the newborn. After childbirth, women cope with the anxiety of embracing a real child for the first time. Furthermore, they have to soothe their baby’s crying often without knowing the cause of crying and ways to soothe it. The occurrence of specific postpartum anxiety was confirmed by no difference in the intensity of other anxieties during this period, for example, the anxiety of getting COVID-19.

The study showed that about 30% of women on the first days after delivery felt NEN and wished to receive support from the staff. Women from the Theo and Drake study [24], who were on the first and the second day after delivery (from 24 h to 77 h), also expressed their temporary willingness to give the newborn to the nursery so they can rest, because their average amount of sleep at night was about 4.4 h. Up to 26% of the respondents from this study reported poor quality of sleep and fatigue, which was influenced by pain, crying of the baby, getting up to breastfeed the baby, and perceived anxiety. In our study, we examined the impact of the COVID-19 pandemic; however, other factors can have a similar effect and result in the lack of support from relatives, e.g., single motherhood, poverty, and illnesses. This allows us to interpret the results of this study in a broader context.

In our study, the NEN in the care of maternity rooming-in staff after CS was greater on both days than the NEN after a natural delivery. In both measurements, in the group who declared having NEN, there were more women after CS than after natural delivery. The above result is consistent with the data collected in the study comparing postpartum fatigue measured in the first 2–3 days after delivery and the difficulties of caring for the baby in women after CS versus natural delivery. Women who gave birth by CS experienced greater postpartum fatigue, which was correlated with greater difficulties with caring for the baby [25].

Our assumption was to examine women in puerperium who had no clinical diagnosis of postpartum haemorrhage and who were already rooming-in women. In the studied population of women in puerperium, the mean decrease in blood haemoglobin concentration was less than 2 g/dL. The analysis showed that there was no significant difference in this parameter between women with and without NEN. No association has also been demonstrated between the drop in the haemoglobin concentration and NEN. The above data allowed us to conclude that the NEN of the women was not dependent on the physical weakness caused by the blood loss after delivery (with M = 1.5 g/dL).

On the other hand, mothers who declared having NEN had a higher level of pain than mothers without such a need. It is common practice in our institution to assess and reduce the patient’s acute somatic pain of the patients according to international recommendations [26] and to investigate its effective alleviation [27]. The motto of the hospital includes the slogan ‘Hospital without pain’. The motto gives the patient the right to inform the staff about subjective, unacceptable levels of pain and the right to relieve it. However, as research in the postpartum period shows, it is impossible to avoid pain completely. A review of the literature conducted by Borges et al. [28] showed that regardless of the analgesic therapy used, more than half of the studied group of 1062 women (52.2%) after CS suffered from severe acute pain during the postoperative period (from 7–10 on the NRS), and 74.1% reported that pain when moving. Another study showed that pain is felt by women regardless of the route of delivery. For a group of 837 women after a natural delivery, average pain scored 3.3 while the worst pain scored 4.9 on the NRS. In a group of 391 women after CS, the average pain level was rated at 4.7, and the worst at 7.1. Both groups reported that postpartum pain affects the performance of daily activities such as movement, ability to concentrate, sleep quality, mood swings, and interactions with others [29]. The results of our study supplemented the above list with the knowledge that the more pain a woman experiences, the greater the NEN. The cut-off point determined in our study by the ROC curve for the identification of women with NEN was 3.5/10 on both the NRS and NRS-A. However, the quality of the NRS-A model was better suited than that of the NRS model. Additionally, the strength of the difference between the levels of anxiety and pain in women with and without NEN was stronger for anxiety (on the NRS-A and STAI-S scale) than for pain. Additionally, it was shown that the higher the state of anxiety (both on the NRS-A and STAI-S scales) the woman experiences, the higher the NEN. In both measurements, the strength of the relationship between NEN and state anxiety was stronger than the strength of the relationship between NEN and acute pain. The results on the differences between women with and without NEN in the levels of specific perinatal anxiety are worth discussing as well. Women who felt NEN had higher levels of perinatal anxiety about self-care for a newborn, breastfeeding, baby crying, and asking staff for help than women without NEN. The fact that the groups did not differ in trait anxiety proves that it was not the higher neuroticism of women with NEN that was associated with anxiety. It could have been influenced by the perception of the specific situation related to self-care in rooming-in. A Turkish study showed that the specific situation of mothers of sick children resulted in a higher level of state anxiety than mothers of healthy children. The groups did not differ in the level of trait anxiety. A difference was obtained between anxiety as a measured state (STAI-S) between a group of mothers whose children were in the newborn intensive care unit and a group of mothers of healthy children [30].

The anxiety of developing COVID-19 had no direct impact on NEN, as no significant difference was found on the second day in the level of anxiety of developing COVID-19 when women with NEN were compared to those without NEN. Indirectly, however, the COVID-19 pandemic changed the situation of women staying at the rooming-in ward, depriving them of support from visitors. The women could only count on the help of the staff. A study by Baran et al. [10] showed that an inadequate level of support from healthcare professionals was a predictor of postpartum depression. Our study showed that the greater anxiety of asking the staff for help from the women can cause the woman to have NEN. The knowledge gained can be used to plan anxiety-reducing interventions to ask staff for help to improve communication between women and staff, which may result in a reduction in NEN.

The above data should be interpreted as the beginning of the development of the research path on the influence of state anxiety and acute pain on NEN of women after delivery. Both acute pain and state anxiety are subjective variables related to a specific moment and situation. The NRS is listed in the Polish recommendation for postoperative pain management in hospitals [31]. There is no recommendation for managing anxiety in hospitals, although data have shown that it has a stronger relationship than pain with the functioning of women in the rooming-in unit in the context of NEN. The easy-to-use numerical NRS-A can be used for routine anxiety measurement. The consistency of the results in all these calculations shows that NRS-A measures dependencies with NEN similarly to the standardized STAI-S scale, which strengthens the conclusions of validation studies based on the comparison of NRS-A with STAI-S, showing NRS-A as a reliable and accurate tool to measure anxiety as a state [21,22].

This study has some limitations that need to be taken into consideration. First, the study did not specify how long the women having NEN would like to leave the newborn under the care of the staff. Estimating the actual amount of time for NEN under staff supervision could open up a further research path. The results of the research on the time of NEN would be the most reliable if women could carry out NEN procedural, and not only declaratively. There was no such system possibility in the present study. The strength of the study was its conduct during the COVID-19 pandemic when all women could count only on the help of staff, not visitors. As a further direction, the NEN can be examined with the participation of visitors in the rooming-in department.

It should also be emphasized that the majority of women in the puerperium (approximately 70%), being without the support of visitors during the COVID-19 pandemic, declared having no NEN. Additionally, in the Theo and Drake study [24], the majority of women after childbirth (60%) expressed positive experiences with the rooming-in system.

## 5. Conclusions

Rooming-in should offer mothers after childbirth a choice of the temporal possibility to rest without a newborn in the room for mothers after delivery, which is not possible in current practice. Further research should be warranted to investigate whether women who give birth in hospitals that meet NEN in the care of maternity staff in their rooming-in units experience less pain and anxiety in comparison to those who give birth in hospital units that do not offer such help for women after childbirth.

## Figures and Tables

**Table 1:** Comparison of the magnitude of the need to entrust the newborn in the care of maternity rooming-in staff by parity and mode of delivery.

Questionnaires	Primiparous		Multiparous			Women after CS		Women after VD		
	Mdn	Mrank	Mdn	Mrank	U (Z)	Mdn	Mrank	Mdn	Mrank	U (Z)
NEN_T1	2.00	105.72	2.00	95.39	1.300	3.00	112.61	2.00	85.09	3.439 **
NEN_T2	2.00	104.62	2.00	96.40	1.044	2.00	108.38	1.00	90.47	2.275 *

CS, Cesarean section; Mrank, mean of ranks in the group; NEN, need to entrust a newborn in the care of maternity rooming-in staff; T1, the first day of puerperium; T2, the second day of puerperium; U (Z), test’s statistics of U Mann–Whitney test; VD, vaginal delivery. * *p* < 0.05; ** *p* < 0.01.

**Table 2:** Relationship between the need to entrust the newborn under the care of staff and anxiety, pain (*n* = 200), and haemoglobin difference (*n* = 198).

Questionnaires	NEN_T1	NEN_T2
Anxiety State (STAI-S)_T1	0.447 **	0.368 **
Anxiety State (NRS-A)_T1	0.439 **	0.332 **
Anxiety State (STAI-S)_T2	0.342 **	0.448 **
Anxiety State (NRS-A)_T2	0.372 **	0.453 **
Fear State (STAI-T)	0.227 **	0.149 *
Pain (NRS_T1)	0.334 **	0.229 **
Pain (NRS_T2)	0.244 **	0.321 **
Haemoglobin difference	0.034	0.133

NEN, need to entrust a newborn in the care of maternity rooming-in staff; NRS, Numerical Rating Scale; STAI, State-Trait Anxiety Inventory; T1, the first day of puerperium; T2, the second day of puerperium. * *p* < 0.05; ** *p* < 0.01.

**Table 3:** Comparison of pain and anxiety of women in relation to the experienced need to entrust the newborn under the care of staff.

Questionnaires	NEN_Low_T1 (*n* = 132)		NEN_High_T1 (*n* = 68)			NEN_Low_T2 (*n* = 146)		NEN_High_T2 (*n* = 54)		
	Mdn	Mrank	Mdn	Mrank	U(Z)	Mdn	Mrank	Mdn	Mrank	U (Z)
NRS	4.00	86.98	5.00	126.74	4.642 **	3.00	92.16	4.00	123.05	3.394 **
STAI-S	40.00	84.58	49.00	131.40	5.421 **	40.00	89.23	48.50	130.97	4.531 **
NRS-A	3.00	84.40	6.00	131.76	5.515 **	3.00	90.28	5.00	128.12	4.135 **
STAI-T	37.00	95.98	39.00	109.26	1.539	37.00	99.41	38.00	103.45	0.439

Mrank, mean of ranks in the group; NEN, need to entrust a newborn in the care of maternity rooming-in staff; NEN_low, women without NEN; NEN_high, women with NEN; NRS, Numerical Rating Scale; STAI, State-Trait Anxiety Inventory; T1, the first day of puerperium; T2, the second day of puerperium; U (Z), test’s statistics of U Mann–Whitney test. ** *p* < 0.01.

**Table 4:** Correlations between the need to entrust the newborn under the care of staff and the specific anxiety on the second day of puerperium.

Specific Postpartum Anxiety Measured on the NRS-A (Score Range 0 to 10)	NEN_T2
Anxiety of self-care for a child during the day	0.459 **
Anxiety of self-care for a child at night	0.516 **
Anxiety of baby crying	0.398 **
Anxiety of natural feeding	0.405 **
Anxiety of asking staff for help	0.382 *
Anxiety of getting COVID-19	0.158 *

NEN, need to entrust a newborn in the care of maternity rooming-in staff; T2, the second day of puerperium. * *p* < 0.05; ** *p* < 0.01.

**Table 5:** Comparison of specific postpartum anxiety between women who felt NEN and those who do not.

Specific Postpartum Anxiety Measured on the NRS-A (Score Range 0 to 10)	NEN_Low_T2 (*n* = 146)		NEN_High_T2 (*n* = 54)		
	Mdn	Mrank	Mdn	Mrank	U (Z)
Anxiety of self-care for a child during the day	1.00	88.76	3.50	132.23	4.793 **
Anxiety of self-care for a child at night	2.00	87.11	5.50	136.71	5.438 **
Anxiety of baby crying	2.00	90.42	4.00	127.74	4.090 **
Anxiety of natural feeding	2.00	90.27	5.00	128.17	4.167 **
Anxiety of asking staff for help	1.00	89.62	3.00	129.92	4.489 **
Anxiety of getting COVID-19	2.00	96.71	4.00	110.74	1.544

Mrank, mean of ranks in the group; NEN, need to entrust a newborn in the care of maternity rooming-in staff; NEN_low, women without NEN; NEN_high, women with NEN; T2, the second day of puerperium; U (Z), test’s statistics of U Mann–Whitney test. ** *p* < 0.01.

## Data Availability

Not applicable.
